# Editorial: Computational Pathology

**DOI:** 10.3389/fmed.2020.00245

**Published:** 2020-06-09

**Authors:** Behzad Bozorgtabar, Dwarikanath Mahapatra, Inti Zlobec, Tilman T. Rau, Jean-Philippe Thiran

**Affiliations:** ^1^École Polytechnique Fédérale de Lausanne (EPFL), Lausanne, Switzerland; ^2^Department of Radiology, University Hospital Center (CHUV), Lausanne, Switzerland; ^3^Inception Institute of Artificial Intelligence, Abu Dhabi, United Arab Emirates; ^4^Faculty of Medicine, Institute of Pathology, University of Bern, Bern, Switzerland

**Keywords:** histopathology images, image analysis techniques, deep learning, clinical outcome analysis, computer-aided diagnosis

The huge amount of information and data available in histopathology images, and ease of their digitization has rapidly advanced the field of computational pathology. The effectiveness of computational pathology, coupled with impressive recent advances in the fields of deep learning and computer vision, provide an ideal setting to revolutionize established clinical workflows.

The goal of this Research Topic is to publish the latest research advances and bring together scientific researchers, medical experts, and industry partners working in the field of computational pathology for clinical outcome analysis.

Emerging technologies such as whole slide imaging (WSI), have increasingly been used to improve the assessment of histological features with several advantages such as easy image accessibility and storage, portability, sharing, annotation, qualitative and quantitative image analysis, and use for diagnostic purposes, including routine diagnosis of liver specimens. The first highlight of this Research Topic is an article entitled “*Whole Slide Imaging and Its Applications to Histopathological Studies of Liver Disorders*,” by Melo et al. This article presents the review of current knowledge on the application of WSI to histopathological analyses of liver disorders as well as to understand liver biology. This article also addresses how WSI may improve the assessment and quantification of multiple histological parameters in the liver, and help diagnose several hepatic conditions with important clinical implications.

The technical limitations of the WSI have further been investigated in the article “*Deep Learning for Whole Slide Image Analysis: An Overview*,” by Dimitriou et al. Building deep learning models capable of understanding WSIs presents novel challenges to the field. This article presents a review work on the interdisciplinary attempt of training deep neural networks using whole slide images, and highlights the different ideas underlying these methodologies.

Another highlight of this edition is an article entitled “*Open Practices and Resources for Collaborative Digital Pathology*,” by Marée. This article describes open practices and open resources in the field of digital pathology with specific focus on approaches that ease collaboration in research and education settings. This review includes open access journals and open peer review, open-source software (libraries, desktop tools, and web applications), and open access collections.

Deep learning algorithms have demonstrated their effectiveness for the segmentation of numerous histological objects (e.g., cell nuclei, glands). However, obtaining annotations for these algorithms is a tedious and potentially biased task. A further highlight of this Research Topic is an article entitled “*Strategies to Reduce the Expert Supervision Required for Deep Learning-Based Segmentation of Histopathological Images*,” by Van Eycke et al.. The article presents a study to review strategies that could help provide the very large number of annotated images needed to automate the segmentation of histological images using deep learning. This review identifies and describes four different approaches: the use of immunohistochemical markers as labels, realistic data augmentation, Generative Adversarial Networks (GAN), and transfer learning.

The imbalance between the increasing demand of highly specialized service and the reduction of specialists able to release this service is a global challenge for pathology. The use of e-pathology devices, such as remote-control microscopes, offers a possible solution to this imbalance. Nano-Eye Device (NED) is a classical microscope integrated by a computer system warranting all the numerous possibilities of an electronic device, including the live control by a distant position. Due to this last characteristic it represents a possible solution for the management of intra-operatory diagnosis in hospitals performing surgical procedures but lacking on-site pathology service. Another highlight of this Research Topic, an article entitled “*Clinical Application of a Real-Time Telepathology System for Frozen Section Diagnosis in Comparison With Optical Microscope*,” by Huang et al.. This article compares the effectiveness of NED to that of optical microscope (OM) in the process of intra-operatory histological diagnosis.

Stain normalization is an important processing task for computer-aided diagnosis (CAD) systems in modern digital pathology. A further highlight of this Research Topic is an article entitled “*A High-Performance System for Robust Stain Normalization of Whole-Slide Images in Histopathology*,” by Anghel et al.. This article addresses two aspects related to the stain normalization pre-processing that is part of modern ML-based pipelines in histopathology. (1) Stain normalization, which can significantly impact the latency of such pipelines especially when dealing with large-size and high-resolution whole-slide images; (2) Poor-quality images can decrease the decision accuracy of the ML pipelines. This article demonstrates that the stain normalization output can be seriously affected when the input image contains artifacts.

Analysis of glandular morphology in H&E stained histopathology slides is among the primary factors in cancer staging and thereby selecting the treatment procedure. So far, deep learning techniques have produced promising results on a few organ-specific gland segmentation tasks, however, the demand for organ-specific gland annotations hinder the extensibility of these techniques to other organs. The last highlight of this edition is an article entitled “*Multi-Organ Gland Segmentation Using Deep Learning*,” by Binder et al.. This article investigates the idea of cross-domain (organ type) gland segmentation strategy that aims at reducing the need for organ-specific annotations. The ability of neural networks based on convolutional layers to isolate and locate this tissue would greatly benefit any specialist aiming to measure gland morphologies within the cancer grading workflow.

All the papers published in this Research Topic underwent peer-review process involving a minimum of two reviewers. We thank the authors for agreeing to publish their papers in this Research Topic, and the reviewers involved in the publishing process of these papers.

## Author Contributions

All authors listed have made a substantial, direct and intellectual contribution to the work, and approved it for publication.

## Conflict of Interest

The authors declare that the research was conducted in the absence of any commercial or financial relationships that could be construed as a potential conflict of interest.

